# Multivariate investigation of *Moringa oleifera* morpho-physiological and biochemical traits under various water regimes

**DOI:** 10.1186/s12870-024-05040-5

**Published:** 2024-06-06

**Authors:** Afef N. Hajaji, Yasmin M. Heikal, Ragaa A. E. F. Hamouda, Mejda Abassi, Youssef Ammari

**Affiliations:** 1 Forest Ecology Laboratory, National Research Institute in Rural Engineering, Water and Forestry, University of Carthage, Bp 10, Ariana, 2080 Tunisia; 2 Botany Department, Faculty of Science, Mansoura University, Mansoura, 35516 Egypt; 3 Department of Biology, Faculty of Sciences and Arts-Khulais, University of Jeddah, Jeddah, Saudi Arabia; 4 Department of Microbial Biotechnology, Genetic Engineering and Biotechnology Research Institute, University of Sadat City, Sadat City, Egypt

**Keywords:** Field capacity, Morpho-physiological parameters, Osmoregulation, Oxidative stress, Drought-resistant tree

## Abstract

**Background:**

The climatic changes crossing the world menace the green life through limitation of water availability. The goal of this study was to determine whether *Moringa oleifera* Lam. trees cultivated under Tunisian arid climate, retain their tolerance ability to tolerate accentuated environmental stress factors such as drought and salinity. For this reason, the seeds of *M. oleifera* tree planted in Bouhedma Park (Tunisian arid area), were collected, germinated, and grown in the research area at the National Institute of Research in Rural Engineering, Waters and Forests (INRGREF) of Tunis (Tunisia). The three years aged trees were exposed to four water-holding capacities (25, 50, 75, and 100%) for 60 days to realise this work.

**Results:**

Growth change was traduced by the reduction of several biometric parameters and fluorescence (Fv/Fm) under severe water restriction (25 and 50%). Whereas roots presented miraculous development in length face to the decrease of water availability (25 and 50%) in their rhizospheres. The sensitivity to drought-induced membrane damage (Malondialdehyde (MDA) content) and reactive oxygen species (ROS) liberation (hydrogen peroxide (H_2_O_2_) content) was highly correlated with ROS antiradical scavenging (ferric reducing antioxidant power (FRAP) and (2, 2’-diphenyl-1-picrylhydrazyle (DPPH)), phenolic components and osmolytes accumulation. The drought stress tolerance of *M. oleifera* trees was associated with a dramatic stimulation of superoxide dismutase (SOD), catalase (CAT), glutathione reductase (GR), ascorbate peroxidase (APX), and glutathione peroxidase (GPX) activities.

**Conclusion:**

Based on the several strategies adopted, integrated *M. oleifera* can grow under drought stress as accentuated adverse environmental condition imposed by climate change.

**Supplementary Information:**

The online version contains supplementary material available at 10.1186/s12870-024-05040-5.

## Introduction

The climatic changes sweeping the world and menace the green life requires urgent attention and the prevention of many disasters, such as the heightened hazard of limited water availability [1]. This horrifying truth that global droughts are increasing over the different regions of world, is driving researchers to focus on finding practical and effective solutions to overcome the intense problematic. In fact, water unavailability is the main limiting factor of plants growth and a total disruption of the several physiological processes causing early senescence and development reduction [2]. As consequence, the hazard effects on field crops largely affected food security [3].

Moringa, together with its 13 species, is one of the most well-known plants studied in science. However, *Moringa oleifera* Lam. is the most studied species, owing to its significant benefits. For the first time, it is crucial to emphasise Moringa’s extraordinary ability to endure drought stress [4]. In addition, Ashutosh et al. [5] in their published update study explain several significant features of this species in detail. Thanks to its safety property for human consumption, the Moringa tree could be considered as promising natural ingredient food product aiming to improve the overall nutritional characteristics [6]. *Moringa oleifera* known as the miracle tree or tree of life, the main representative of the order Moringa, it belongs to family Moringaceae and it is also commonly known as horseradish tree or drum stick. Moringa has both medicinal and nutritional values and uses with essential minerals, amino acids and vitamins [7]. Especially, leaves were largely used because of their high tenor of proteins, essential amino acids, dietary fibres, vitamins, and minerals, while being low in fat, sugars, and total starch protein and total dietary fibre amounts [8].

Moringa is referenced for its diverse therapeutic properties, for example antipyretic, antipathy to diabetes, calming, anticancer and antiulcer [9]. Seeds oils, which are rich in bioactive compounds, were used as anti-inflammatory, nephroprotective, antioxidant, anti-diabetic, anti-cancer, and anti-obesity [10]. In the agronomic sector, Moringa can be used as biofertilizer reducing the costs of chemical fertilizer application and save the environment. Several studies realised by Yap et al. [11], Yaseen and Takacs-Hajos [12], Chuene et al. [13] showed that Moringa used as fertilizer, improve crops productivity of *Foeniculum vulgare*, Mill, *Solanum lycopersicum*, *Silybum marianum, and Lactuca sativa* under normal and stress conditions.

Face to the environmental horrible problem, many plant species tolerate drought stress and survive under limited water availability. Moringa is one of the miraculous plants that incorporate several ecophysiological mechanisms to persist face this abiotic stress factor [14, 15]. Under stress conditions, plants present several strategies to overcome the negative induced effect produced through production of large range of chemicals [16]. Secondary metabolites were generated to support the internal changes caused by stress factors. More precisely, they play an antioxidant role through hydrogen donation from phenolic groups to quenching of free radicals [17]. Drought stress which becoming more erratic, caused significant changes in nutritional quality and biochemical compounds such as phenolic acids [18].

Alternatively, in stressed plants, the build-up of appropriate organic solutes (betaines, soluble sugar, proline, and organic acids) is observed to preserve cellular homeostasis, shield enzymes, and scavenge reactive oxygen species [19]. Reactive oxygen species (ROS) generation is mainly attributed to the negative effect of stress conditions. To endure this oxidative damage and to improve their antioxidant potential, plants cells modulate several enzymatic responses. Antioxidant enzymes (superoxide dismutase (SOD), catalase (CAT), Ascorbate peroxidase (APX) and glutathione reductase (GR) peroxidase (POD) were stimulated under abiotic stress such as, salinity and drought. In fact, numerous studies [15, 20] showed a positive correlation between abiotic stress factors, bioactive molecules accumulation such as polyphenols and the enzymatic antioxidant activities enhancement in *Moringa oleifera* in order to mitigate the unfavourable conditions of growth.

To address the lack of water availability effects, this study was conducted to: (1) assess the morphophysiological parameters, growth responses, secondary metabolites, osmoregulators, and enzymatic and non-enzymatic antioxidant changes in *Moringa oleifera* trees. (2) Use various multivariate analysis approaches to verify and validate the relationships between morphophysiological parameters and biochemical responses.

## Materials and methods

### Plant material and growth conditions

*Moringa oleifera* Lam. seeds collected from Bouhedma National Park (Tunisian center, east) in 2018, were cultivated in the research area at the National Institute of Research in Rural Engineering, Waters and Forests (INRGREF) of Tunis (Tunisia) (latitude: 36° 50ʹ North, longitude: 10° 14ʹ East, altitude: 3 m). The resulting three years old trees were transferred to pots filled with 9 kg of soil (mixture of sand and loamy soil (1/4)). The experiment was conducted for 60 days (July-August 2021) in a completely randomized design with different field capacities (25, 50, 75, and 100%) and three replicates. The weather in July and August 2021 in the experimental location was characterized, respectively by an average of 28 and 29 °C, in the early morning, 34 and 36 °C at midday, and 41 and 45 °C at the end of the day. The temperature was recorded at 41 and 45 °C in July and August, respectively (https://www.historique-meteo.net).

### Growth parameters

The height of the plant, diameter of the principal stem, and leaves parameters (Fresh weight, dry weight, area, length, width, and perimeter) were determined at t_0_ (3 years aged trees), t_1_ (after 15 days), t_2_ (after 30 days), t_3_ (after 45 days) and t_4_ (after 60 days), in plants grown under different drought stress conditions. The different leaf parameters were measured using a planimeter (model CI-202, USA). At the end of the experimental period, five separate plants subjected to the different field capacities were used to determine leaves, stems, and roots’ fresh weight, dry weight, length, diameter, and fluorescence measurements. Dry weight was determined following drying in an oven at 60 °C for 72 h.

Relative growth rate (RGR, mg. g^− 1^. d^− 1^) was determined following equation:

$$(ln{w}_{4}-ln{w}_{0})/({t}_{4}-{t}_{0})$$

*w*_*0*_ and *t*_*0*_ are the shoot weight and time at the beginning of the experiment, and w_4_ and t_4_ were shoot weight and time at the end of the experiment, respectively.

Net assimilation rate, (NAR, g. m^–2^. d^–1^) was calculated following the following equation:

$$({w}_{4}-{w}_{0})/dt\ast(ln{LA}_{4}-ln{LA}_{0})/({LA}_{4}-{LA}_{0})$$

*w*_*0*_ and *w*_*4*_ are the dry weight of biomass; *dt* per unit time; *LA*_*0*_ and *LA*_*4*_ are the total leaf area and t_0_ and t_4_ are the time at the beginning and the end of the experiment, respectively.

Relative leaf area expansion rate, (RGRA, mm^− 2^.d^− 1^) was determined following the equation:

$$(ln {LA}_{4}\hspace{0.17em}-\hspace{0.17em}ln {LA}_{0})/({t}_{4}\hspace{0.17em}-\hspace{0.17em}{t}_{0})$$

*LA*_*0*_ and *LA*_*4*_ are the total leaves area; t_0_ and t_4_ are the time at the beginning and the end of the experiment, respectively.

The relative growth rate of the principal stem in height (RGRh, cm. cm^–1^.d^–1^) was calculated according to the following formula:

$$(ln{h}_{4}-ln{h}_{0}/{t}_{4}\hspace{0.17em}-\hspace{0.17em}{t}_{0})$$

*h*_*0*_ and *h*_*4*_ are the stem height; t_0_ and t_4_ are the time at the beginning and end of the experiment, respectively.

### Determination of relative chlorophyll content and fluorescence measurements

The relative chlorophyll concentration (SPAD) was evaluated at the end of treatments using the chlorophyll meter (model SPAD-502, Minolta, Japan). The measure of the maximum quantum efficiency of PSII photochemistry (Fv/Fm) was assessed in the same leaves after 30 min. of dark adaptation, using modulated chlorophyll fluorometer (OS1p, Opti-Sciences Inc, Hudson, USA).

### Determination of MDA and H_2_O_2_ contents

The lipid peroxidation level of foliar tissues was determined as MDA (Malondialdehyde). To do, concentration of thiobarbituric acid-reacting substances (TBARS) was measured spectrophotometrically at 535 nm, as described by Alia et al. [21].

The content of hydrogen peroxide (H_2_O_2_) in leaves was determined based on the modified method of Patterson et al. [22]. Colorimetric method (λ = 508) was used to determine H_2_O_2_ content, using H_2_O_2_ as a standard.

### Antioxidant activity: FRAP (ferric reducing antioxidant power) and DPPH (2, 2’- diphenyl-1- picrylhydrazyle) radical-scavenging activity assays

Hinneburg [23] method was used to realise FRAP test. Leaves extracts were incubated (At 50 °C for 30 min) in presence of phosphate buffered saline PBS and K_3_Fe (CN)_6_. After that, Trichloric acid (10% TCA) and FeCL_3_ (0.1%) were added, respectively in which the reduction of ferric ion (Fe^3+^)-ligand complex to the brightly blue ferrous (Fe^2+^) complex by antioxidants in an acidic solution. 30 min. later, optical density was determined at 700 nm. Results were expressed in mmol ascorbic acid equivalent per gram of dry extract (mmol AAE/g).

DPPH anti-radical activity was carried out according to Parejo et al. [24] proceed. Each foliar extract (at different concentrations) was incubated with DPPH reactive for 30 min. To determine DPPH reduction, the absorbance of the DPPH solution as well as samples were measured at 517 nm. These optical densities were used for determination of sample concentration needed to neutralize 50% of radicals (IC_50_).

### Total phenolic, total flavonoids, and condensed tannins contents quantification

Extraction of secondary metabolites was realised by maceration (methanol, 70%) for 48 h. After filtration on Whatman paper No.1, the filtrates obtained were evaporated at using a rotary evaporator at 60 °C. The residues of this filtrate were dried in an oven for 48 h at 45 °C to obtain the extracts dry.

Total phenolic content was measured using the Folin–Ciocalteu method described by Li et al. [25]. Total phenolic content was expressed as gallic acid mg. g^− 1^ dry weight (mg GAE.g^− 1^). Flavonoid content was carried out according to the method of Dehpeur et al. [26]. Contents were expressed in mg quercetin equivalent per gram of dry weight (mg Qu. g^− 1^). Total condensed tannins were estimated according to Ba et al. [27]. Contents were expressed in mg catechol equivalent/g dry weight (mg CAT. g^− 1^).

### Proline and soluble sugars contents determination

Proline analysis was performed according to the protocol described by Bates [28] and expressed as µmol. g^− 1^ DW. Each extract was added to glacial acetic acid and Ninhydrin reagent. The mixture was placed in water bath at 100 °C for 30 min. After that, toluene was added, and the absorbance was determined spectrophotometrically at 520 nm.

Total soluble sugars were fixed according to Dubois et al. [29] method. Contents were estimated by Anthrone reagent and defined as µmol. g^− 1^ DW. The mixture was placed in water bath (100 °C) for 10 min. The absorbance was spectrophotometrically measured at 630 nm.

### Antioxidant enzymatic extraction and assays

Enzyme extractions were carried out at 4 °C. The fresh plant tissues were powdered in liquid nitrogen and extracted at a ratio of 1:3 (w/v) fresh weight. Protein content was determined by Bradford’s method [30] using bovine serum albumin (BSA) as the standard. SOD; superoxide dismutase (EC: 1.15.1.1) activity was determined by the photochemical method according to Beyer and Fridovich [31] (1987). Catalase (CAT; EC: 1.11.1.6) activity assay was recorded following the method of Cakmak [32]. Ascorbate peroxidase (APX; EC: 1.11.1.11) activity was measured according to Nakao and Asada [33] by monitoring the rate of ascorbate oxidation at 290 nm (E = 2.8 mM^− 1^ cm^− 1^). Glutathione reductase (GR; EC: 1.6.4.2) activity was determined by monitoring the GSH-dependent oxidation of NADPH at 340 nm according to Rao et al. [34]. Peroxidase (GPX; EC: 1.11.1.9) activity was measured by the increase in absorbance at 470 nm caused by guaiacol oxidation according to Decleire et al. [35].

### Statistical analysis

All data were subjected to one way ANOVA analysis based on Tukey’s HSD test (IBM SPSS Statistics 23) to determine the significant differences between means of treatments at a probability level of 0.05. All data were evaluated for normality using Shapiro-Wilk at the 0.05 level prior to statistical analysis, and if needed, the data were transformed. A randomised complete block design with three replications was used to gather the data. Every parameter under study was given its mean (± standard deviation). For the graphical representation of morpho-physiological characteristics, the JMP® ver. 17.2.0 cell plot was employed (SAS Institute Inc., Cary, NC, USA, 2022–2023). Principal Component Analysis (PCA) was used to examine the contribution of the combination of morpho-physiological, biochemical, and metabolic characteristics to better summarize the data and comprehend the conclusions. For additional analysis, components with an Eigen value larger than one were retained. Scatter plot matrix with heatmap correlation circles of 29 quantitative traits were calculated. The similarity matrix was produced by employing the hierarchical co-clustering (dendrogram) utilizing the ward method and a heatmap based on all the data. These studies were performed with JMP® version 17.2.0.

## Results and discussion

### Morpho-physiological traits

**Fig. 1:**
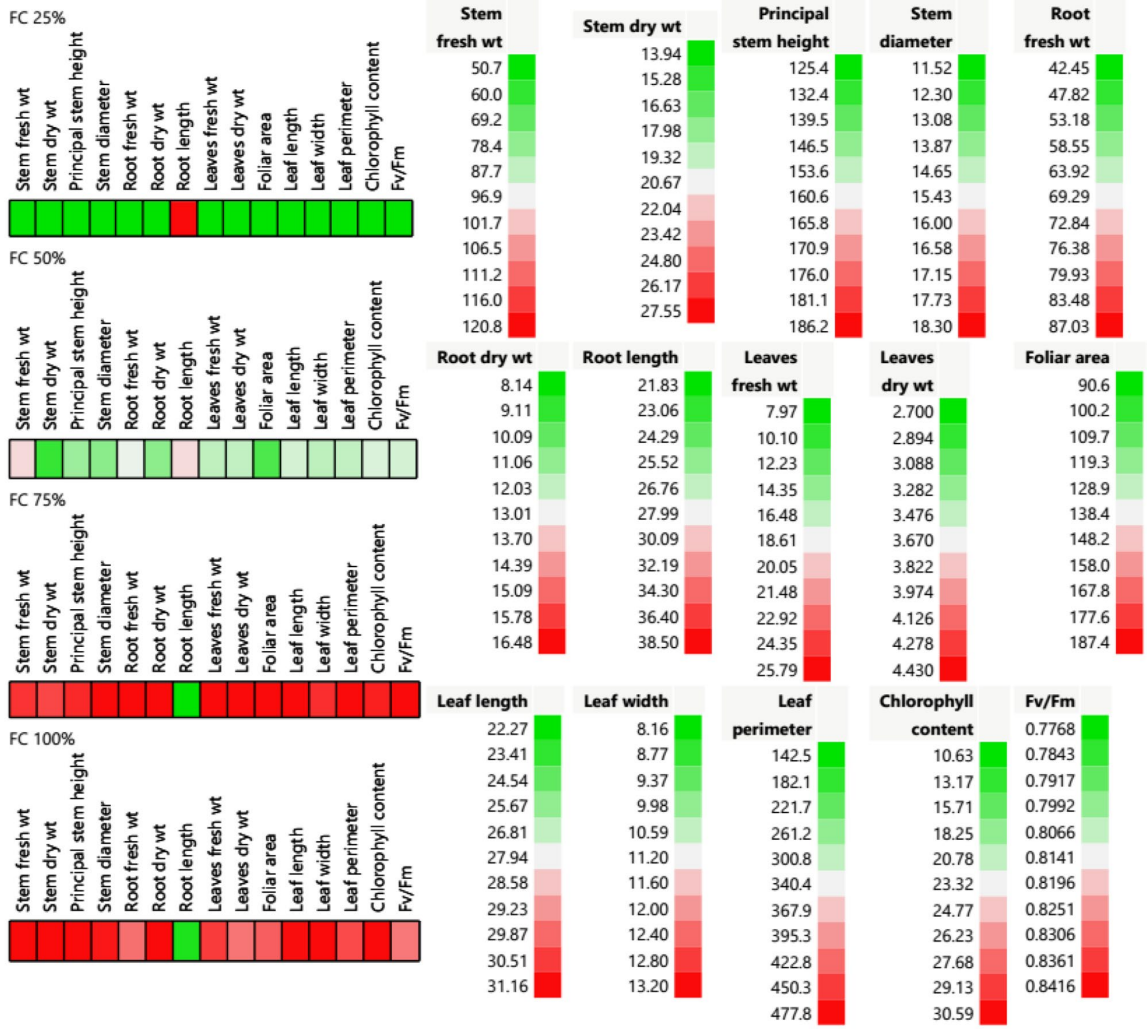
Cell plot of 15 morpho-physiological traits of stem, root, leaf parameters, relative chlorophyll content and fluorescence measurements (Fv/Fm efficiency) of *Moringa oleifera* trees submitted to different field capacities (25, 50, 75, and 100%). Red color assumes the highest value, while the green color assumes the lowest ones

Abiotic stresses such as drought, affect negatively every growth stage of many crops by reducing leaf production, photosynthesis, and yield [36]. Cell plot results of 15 quantitative morpho-physiological traits showed tremendous variations of *M. oleifera* trees under different field capacity regimes (Fig. 1**).** An analogous trend in the root length of Moringa plants was noted. Under the extreme field capacity (25% FC), all the quantitative traits showed the lowest values, which marked in green, in different tissues (stem, root and leaves) except root length parameter which recorded the highest value (remarked as red). Also, under 50% FC, all *M. oleifera* parameters showed intermediate color and values except stem fresh weight and root length parameters obtain had values near maximum and remarked as faint red color. In the contrast, under 75 and 100% showed the highest values of all parameters which marked with red color and root length (remarked with green) showed the contrary value of the tested parameters of 25% FC as illustrated in **Fig. (1)**.

On the other hand, data showed a reduction of the shoot and roots’ fresh and dry weights (Supplementary Fig. 1), principal stem height, and diameter under accentuating situation of water stress (25% FC). In contrast, a dramatic enhancement of root length was noted in trees submitted to 25% FC (Supplementary Fig. 1G). The impact of water unavailability on *M. oleifera* growth and development was reflected by other morphological parameter changes. When low water availability was applied in a culture medium (25%), the shorter plant height and less stem diameter were recorded (Supplementary Fig. 1E and F).

This regression of height and diameter was the results of cells elongation, turgor, and volume reduction as described by Heng et al. [37] and Ximeng et al. [38]. More that, slower organs growth under stress condition is a strategy adopted to acclimate to the stress conditions [38]. In contrast, when the water amount was very limited (25% FC), roots are longer compared to those in control plants (Supplementary Fig. 1G). This responsible absorption water organ showed a more developed expansion and proliferation attributed to the necessity to search and acquisition of water in the soil under low osmotic pressure. Also, the morphological and traits changes of roots, explain the Moringa tree orientation to a more water and nutrients conservative strategy.

Far, no senescence symptoms were observed in all our trees subjected to the experimental protocol. For this reason, we have measured the maximum quantum efficiency of PSII (Fv/Fm) in Moringa leaves at the end of the experiment period. We found that this parameter was reduced by water lack (Supplementary Fig. 1H). In fact, PSII efficiency was under regulation of the quantum yield (Fv/Fm) to predicts plant development under various environmental conditions [39]. It is true that the decrease of PSII efficiency caused by abiotic stress resulted in photoinhibition and cause photodamage [14, 40]. However, in another way, the diminution of Fv/Fm is another benefit response presented by plants to minimize photosynthetic activity under stress factor [39].

Crop plants exhibit a variety of drought stress adaptation and acclimatization mechanisms, ranging from ostensibly straightforward physiological or morphological characteristics that function as critical stress tolerance [41]. To encompass, we can suggest that the origin of tolerance capacity to water stress of *M. oleifera*, is identified by the maintenance of clear photosynthetic assimilation at the level of the leaves, monitoring height of shoot part, and enhancement of roots elongation.

### Growth response

**Fig. 2:**
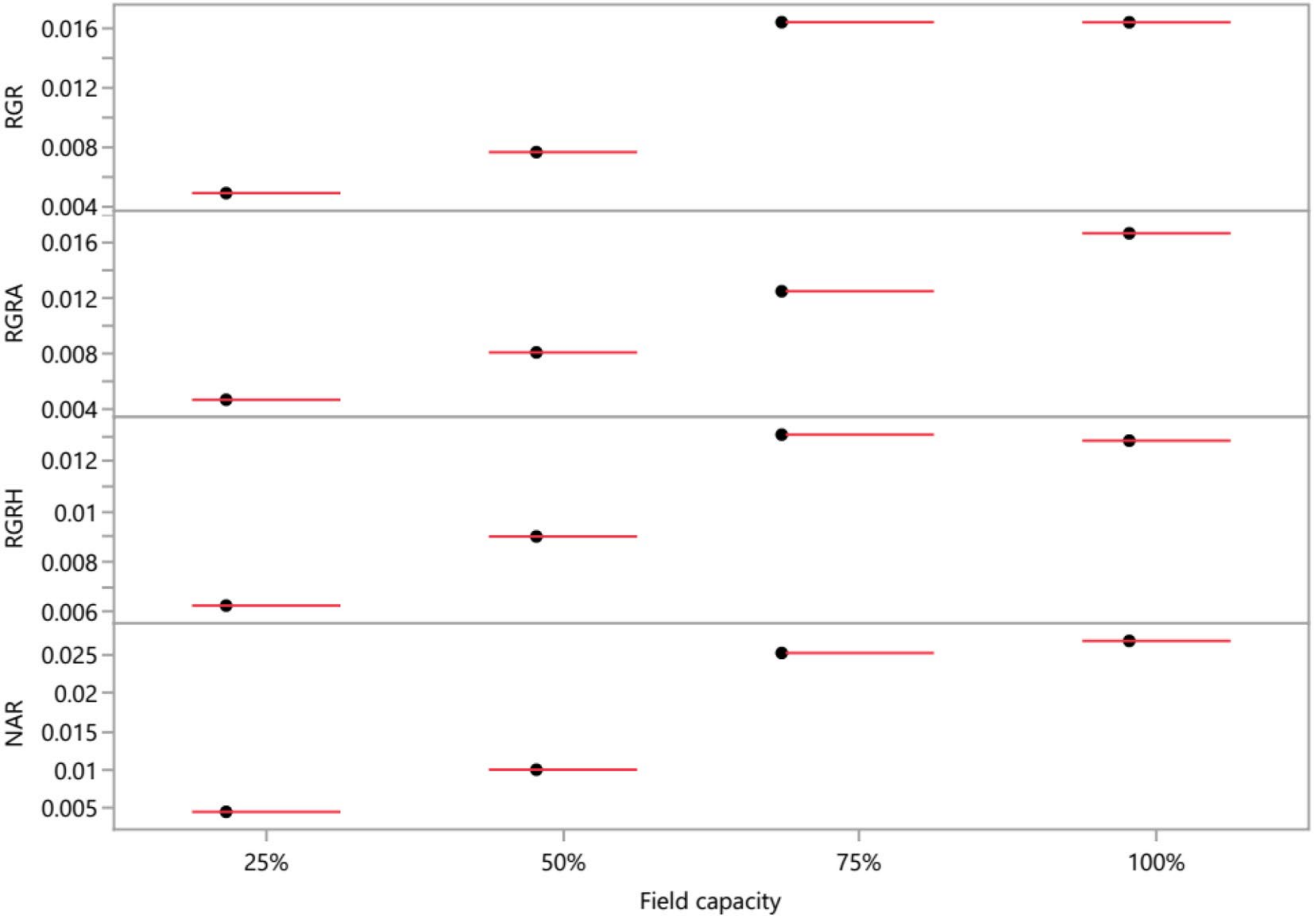
Scatter plot matrix of variation in the cumulative average of final growth rates (%): Relative growth rate (RGR mg. g^− 1^.d^− 1^), Relative leaf area expansion rate, (RGRA mm^− 2^.d^− 1^), Relative growth rate of the principal stem in height (RGRh, cm. cm^–1^.d^–1^) and Net assimilation rate, (NAR g. m^–2^.d^–1^) of *M. oleifera* trees submitted to different field capacities (25, 50, 75 and 100%)

Several key indices were determined as an aid to understanding the growth response of *M. oleifera* trees face to the different water-holding capacities applied during the experiment period. In the beginning, relative growth rate (RGR) was determined, because it is useful for analyzing whole plant response to the abiotic stress factor fixed in this study (Fig. 2). Under severe water unavailability (25% FC), RGR is in order of 0.016 mg. g^− 1^d^− 1^, which showed 70% decline compared with the positive control (100%) (Supplementary Fig. 2A, E). Secondly, these results showed that the relative leaf expansion (RGRA) (Fig. 2B, E) was considerably reduced, especially in plants subjected to water restriction (25% FC) by − 71.78%. This may explain, first, the drop in net biomass gains which presents the result of the assimilation of CO_2_ by the leaves minus the respiratory loss by the whole plant. Indeed, the net CO_2_ assimilation rate (NAR) was found to be reduced by (0.01 and 0.004 g^− 1^. d^− 1^) which represented the declining percentage of 62.7 and 83.2, respectively when the plants were grown at field capacities of 50 and 25% (Fig. 2C, E). For further evidence, it was also found that the relative growth rate in height (RGRh) of the principal stem was inhibited by 51.33% from 0.004 mm^− 1^. d^− 1^ (control condition) to 0.002 mm^− 1^. d^− 1^ under severe drought conditions (25% FC) (Fig. 2D, E). The link between growth modifications and interior leaf biological (cell division and expansion) and physiological (photosynthesis) processes under conditions of water deprivation can help us to understand these findings. Numerous authors have correctly pointed out that leaves primarily regulate a plant’s ability to withstand drought [38].

To confirm this previous suggestion, leaf dimensions, which are the major driving variables for photosynthetic machinery and whole-plant growth, were followed and measured for 60 days of applied drought stress. Table 1 demonstrated that there was a non-significant difference in the various leaf measurements between 75% and 100% FC at *P* ≤ 0.05 level. Whereas, compared to the control condition, a 25% FC provoked a net reduction of these parameters at the four times (t_1_, t_2_, t_3_ and t_4_) set for taking the measurements. We found that, after 60 days (t_4_), extreme field capacity (25% FC) induced reduction of leaf fresh (-47.07%) and dry weights (-37.34%), area (-53.4%), length (-67.34%), width (-43.93%), and perimeter (-51.77%). These leaves’ morphological adjustments generated by the water constraint condition, resulted in growth performance and adaptability to this stress factor [37, 39, 40]. The limitation in leaves expansion resulting in stomatal closure is an adaptative tolerance mechanism included in photosynthetic machinery protection under such environmental condition.

### Antioxidant activity, membrane lipid peroxidation, secondary metabolites and osmolytes contents

The ability of antioxidants to scavenge free radicals is measured by the 2,2 diphenyl-b picrylhydrazyl (DPPH) radical scavenging activity assay [42]. It was revealed that FRAP and DPPH assays are often used to evaluate the ability of the extract to scavenge free radicals produced because of the induced-oxidative stress biological damage [15]. The compound’s ability to stop b-carotene from oxidising was determined by Chitiyo et al. [43] using the b-carotene-linoleic acid model system, which demonstrated a higher antioxidant capacity in *M. oleifera* (75% FWC) under mild stress. Our agreed data showed that the highest total antioxidant activity (FRAP and DPPH) and radical scavenging activity corresponding to the lowest IC_50_ value was marked in leaves extract derived from plants grown under the reduced capacity field (25% FC) (Table 2). Furthermore, these same plants demonstrated the greatest ability to scavenge ROS. The variability observed in the relationship between polyphenolic compounds and antioxidant capacities as indicated by the different antioxidant measurements may be attributed to the fact that biological activity is influenced by the elevated concentration of total secondary metabolites (phenolic compounds) [44].

**Table 1:** Kinetic measurements of leaf growth parameters and chlorophyll content of *Moringa oleifera* trees submitted to different water potentials

Parameter	Treatment (Field capacity %)	Experiment time course				
		t_0_	t_1_	t_2_	t_3_	t_4_
Leaf fresh weight (g)	25%	2.007 ± 0.084^a^	2.11 ± 0.01^c^	2.178 ± 0.137^c^	2.180 ± 0.11^c^	1.901 ± 0.012^c^
	50%	2.007 ± 0.084^a^	2.33 ± 0.014^b^	2.504 ± 0.134^b^	2.808 ± 0.18^b^	2.911 ± 0.030^b^
	75%	2.007 ± 0.084^a^	3.015 ± 0.030^a^	3.202 ± 0.207^a^	3.300 ± 0.25^a^	3.538 ± 0.110^a^
	100%	2.007 ± 0.084^a^	3.002 ± 0.020^a^	3.197 ± 0.255^a^	3.3300.28 ± ^a^	3.592 ± 0.271^a^
Leaf dry weight (g)	25%	0.152 ± 0.010^a^	0.170 ± 0.051^d^	0.185 ± 0.01^d^	0.193 ± 0.01^d^	0.203 ± 0.01^d^
	50%	0.152 ± 0.010^a^	0.192 ± 0.07^c^	0.198 ± 0.01^c^	0.207 ± 0.013^c^	0.274 ± 0.014^c^
	75%	0.152 ± 0.010^a^	0.284 ± 0.013^a^	0.301 ± 0.024^a^	0.325 ± 0.01^b^	0.335 ± 0.025^b^
	100%	0.152 ± 0.010^a^	0.261 ± 0.022^b^	0.267 ± 0.021^b^	0.301 ± 0.027 ^a^	0.324 ± 0.03^a^
Leaf water content (g.g^− 1^DW)	25%	12.380 ± 1.02^a^	12.170 ± 0.98^c^	10.301 ± 1.02^c^	9.499 ± 0.08^c^	7.525 ± 0.63^c^
	50%	12.380 ± 1.02^a^	12.281 ± 1.00^b^	11.235 ± 1.00^b^	10.643 ± 1.18^b^	9.990 ± 0.85^b^
	75%	12.380 ± 1.02^a^	12.391 ± 1.55^a^	12.508 ± 0.98^a^	12.50 ± 0.96^a^	12.501 ± 1.00^a^
	100%	12.380 ± 1.02^a^	12.3891 ± .03^a^	12.490 ± 1.100^a^	12.495 ± 1.03^a^	12.495 ± 1.3^a^
Leaf area (cm^2^)	25%	140.788 ± 8.32^a^	138.95 ± 12.22^c^	133.617 ± 10.1^c^	128.878 ± 9.84^c^	116.959 ± 9.06^c^
	50%	140.788 ± 8.32^a^	148.397 ± 9.64^b^	156.024 ± 13.1^b^	161.097 ± 9.66^b^	193.965 ± 10.7^b^
	75%	140.788 ± 8.32^a^	200.072 ± 13.00^a^	241.600 ± 19.06^a^	245.86 ± 10.23^a^	250.302 ± 18.3^a^
	100%	140.788 ± 8.32^a^	199.318 ± 10.6^a^	240.727 ± 12.6^a^	246.79 ± 15.64^a^	251 ± 11.4^a^
Leaf length (cm)	25%	26.439 ± 1.13^a^	24.958 ± 2.140^c^	50.362 ± 2.71^c^	50.038 ± 3.21^c^	51.303 ± 4.58^c^
	50%	26.439 ± 1.13^a^	32.503 ± 3.010^b^	55.342 ± 3.5^b^	77.749 ± 6.55^b^	108.334 ± 7.62^b^
	75%	26.439 ± 1.13^a^	37.500 ± 3.000^a^	86.000 ± 0.82^a^	104.77 ± 8.76^a^	159 ± 13.08^a^
	100%	26.439 ± 1.13^a^	37.405 ± 2.700^a^	85.712 ± 6.6^a^	106.072 ± 8.71^a^	157.076 ± 12.2^a^
Leaf width (cm)	25%	9.969 ± 0.570^a^	10.115 ± 1.050^c^	10.257 ± 0.852^d^	12.029 ± 0.97^c^	14.62 ± 1.3^c^
	50%	9.969 ± 0.570^a^	10.375 ± 0.970^b^	12.362 ± 1^c^	15.538 ± 1.23^b^	19.105 ± 1.36^b^
	75%	9.969 ± 0.570^a^	12.725 ± 1.110^a^	15.887 ± 1.09^b^	21.04 ± 1.22^a^	26.781 ± 1.08^a^
	100%	9.969 ± 0.570^a^	12.708 ± 1.020^a^	17.4 ± 1.07^a^	20.258 ± 1.05^a^	26.075 ± 1.37^a^
Leaf perimeter (cm)	25%	252.02 ± 18.1^a^	329.64 ± 29.7^c^	303.045 ± 25.4 ^c^	250.194 ± 17.2 ^c^	264.791 ± 14.5 ^c^
	50%	252.02 ± 18.1^a^	357.792 ± 30.10^b^	380.421 ± 26.30 ^b^	335.49 ± 22.17 ^b^	368.201 ± 20 ^b^
	75%	252.02 ± 18.100^a^	375.187 ± 27.30^a^	467.918 ± 22.10 ^a^	510.00 ± 33.080 ^a^	545.78 ± 23.71 ^a^
	100%	252.02 ± 18.100^a^	374.08 ± 25.100^a^	468.018 ± 36.2^a^	508.947 ± 25.5^a^	549.106 ± 28.6^a^
**Chlorophyll content** **(nmol.g** ^**− 1**^ **FW)**	25%	20.645 ± 1.84^a^	20.682 ± 0.990 ^c^	20.126 ± 1.640 ^c^	16.130 ± 0.990 ^c^	10.633 ± 0.88^c^
	50%	20.645 ± 1.84^a^	22.330 ± 1.050 ^b^	23.806 ± 1.410 ^b^	23.288 ± 1.060 ^b^	22.061 ± 1.06^b^
	75%	20.645 ± 1.84^a^	23.188 ± 9.650^a^	25.77 ± 2.030 ^a^	30.020 ± 1.400 ^a^	30.000 ± 2.81^a^
	100%	20.645 ± 1.84^a^	23.666 ± 10.960^a^	25.252 ± 1.200 ^a^	30.141 ± 2.810 ^a^	30.586 ± 2.07 ^a^

One way ANOVA represented by means ± (deviation of three measurements). Different superscript letters showed statistically significant values (according to Tukey’s HSD test) at *P* ≤ 0.05. *t* is the time, *t*_0_ (3 years aged trees), t_1_ (after 15 days), t_2_ (after 30 days), t_3_ (after 45 days) and t_4_ (after 60 days)

**Table 2:** Antioxidant activities (enzymatic and non-enzymatic), membrane lipid peroxidation, secondary metabolites and osmolytes contents of *M. oleifera* submitted to different field capacity regimes

Biochemical parameter	Treatments (Field capacity%)			
	25%	50%	75%	100%
MDA (nmol. g^− 1^ DW)	375.236 ± 39.681^a^	232.366 ± 15.642^b^	116.200 ± 9.63 ^c^	97.023 ± 8.05 ^d^
H_2_O_2_ (nmol. g^− 1^ FW)	53.746 ± 4.51^a^	32.560 ± 2.145 ^b^	18.127 ± 1.46 ^c^	19.163 ± 1.08 ^cd^
FRAP (mmol AAE/100 g)	87.440 ± 7.91^a^	55.240 ± 3.57 ^b^	26.010 ± 2.03^c^	24.740 ± 1.07 ^cd^
DPPH (µg/mL)	4.820 ± 0.025^d^	8.690 ± 0.63 ^c^	12.570 ± 1.01^b^	13.070 ± 0.913^ab^
Total phenols (mg eqAG.g^− 1^DW)	64.180 ± 0.518 ^a^	38.120 ± 1.38 ^b^	11.970 ± 0.11 ^c^	9.340 ± 0.084 ^d^
Flavonoids (mg eq Qu.g^− 1^DW)	23.451 ± 0.119 ^a^	9.911 ± 0.0746 ^b^	4.556 ± 0.048 ^c^	3.710 ± 0.031 ^d^
Tannins (mg eq catch. g^− 1^DW)	2.554 ± 0.133 ^a^	1.155 ± 0.013 ^b^	0.750 ± 0.907 ^c^	0.838 ± 0.087 ^d^
Proline (µmol. g^− 1^DW)	17.472 ± 1.07 ^a^	13.153 ± 0.857 ^b^	10.791 ± 0.932 ^c^	8.669 ± 0.61 ^d^
Soluble sugar (µmol. g^− 1^DW)	16.241 ± 0.843 ^a^	12.573 ± 1.05 ^b^	10.156 ± 0.974 ^c^	8.125 ± 0.651 ^d^

One way ANOVA represented by means ± (deviation of three measurements) showing different statistically significant superscript letters (according to Tukey’s HSD test) at *P* ≤ 0.05. Malondialdehyde (MDA); hydrogen peroxide (H_2_O_2_); Total antioxidant activity FRAP (ferric reducing antioxidant power); DPPH (2, 2’- diphenyl-1- picrylhydrazyle) radical-scavenging activity

More than that, a positive relationship was observed between the FRAP and DPPH activities increase, and MDA and H_2_O_2_ accumulation at the different water holding capacities. Increasing drought stress levels carries the antioxidative potential for chain-breaking inhibition of lipid peroxidation scavenging as previously suggested by Nasraoui et al. [45] and Abdul Waheb et al. [19]. On the other hand, the high antioxidant capacity detected may be attributed to the cooperative effects of antioxidant activity (DPPH) and high total phenolic, flavonoids, and tannins contents (Table 2).

According to McMahon et al. [46], condensed tannins are a product of flavonoid polymers. Popovic et al. [47] found that drought-induced slightly greater amounts of condensed tannins were found in poplar trees. Moreover, transgenic poplar lines with high condensed tannin content showed decreased levels of hydrogen peroxide, photosystem II damage, and leaf death [48]. This makes sense since it demonstrates a clear link between high tannin content and antioxidant capacity.

Furthermore, phenolic compounds contents of edible plants may be considered an inducement of their antioxidant capacity as showed by several researchers [13, 15–17], phenolics compounds have been shown to act as a photoprotector by blocking the stimulation of chlorophyll under conditions of moisture stress by decreasing light absorption [49]. The second harvest of *Moringa oleifera* under extreme stress had the highest concentrations of total phenolic compounds (35% FWC) and proanthocyanidins (50%) for flavonoids [43].

Osmotic stress and the other different abiotic stress factors (salinity, metallic) induce the synthesis and accumulation of osmoregulator molecules such as proline amino acid and soluble sugar [50]. It is largely demonstrated that proline participates in reinforcement of the antioxidant system scavenging induced ROS accumulation [19]. According to our data, Chitiyo et al. [43] found that in drought stressed *Moringa* proline and soluble sugar contents increased with the severity of moisture stress in all plant parts (root, stem and leaf), with the leaves under severe stress (35% FWC) recording the highest concentration. A strong correlation of these compounds’ accumulation and higher radical scavenging activity against FRAP and DPPH radical were detected confirming their role in antioxidant defense potential of Moringa [15, 51]. This corroborates the results of the present investigation, which demonstrated a positive correlation between antioxidant activity, secondary metabolites and osmolytes amounts. In addition to having the highest concentrations of condensed tannins and total phenolic compounds which are known to rise with an elevation in phenolic concentration in the severely stressed (25% FC) plants also showed the highest proline and soluble sugar tenors ferric reducing capability. This suggests that *M. oleifera*’s antioxidant properties are enhanced by moisture stress. By raising the amounts of antioxidant substances such as total phenols, and flavonoids, drought stress improved the therapeutic potentials of Moringa. In fact, free radicals can be chelated by total phenolics and flavonoids before they damage cells [52]. Briefly, *M. oleifera* drought tolerance is achieved through metabolite pattern alteration, which ensures good cellular activities.

### Antioxidant enzyme activity

The crucial role in the reduction of reactive oxygen species generation and resistance to oxidative damage by activation of a defensive antioxidant system in stressed plants is largely confirmed in other studies [44, 50]. The stimulation of antioxidant enzymes (SOD and CAT) to react more strongly to freshwater deficiency stress was triggered by the first water deficit cycle in control stressed plants. These findings suggest that these plants are best protected in the peroxisome, where CAT breaks down H_2_O_2_ produced by photorespiration [53].

**Fig. 3:**
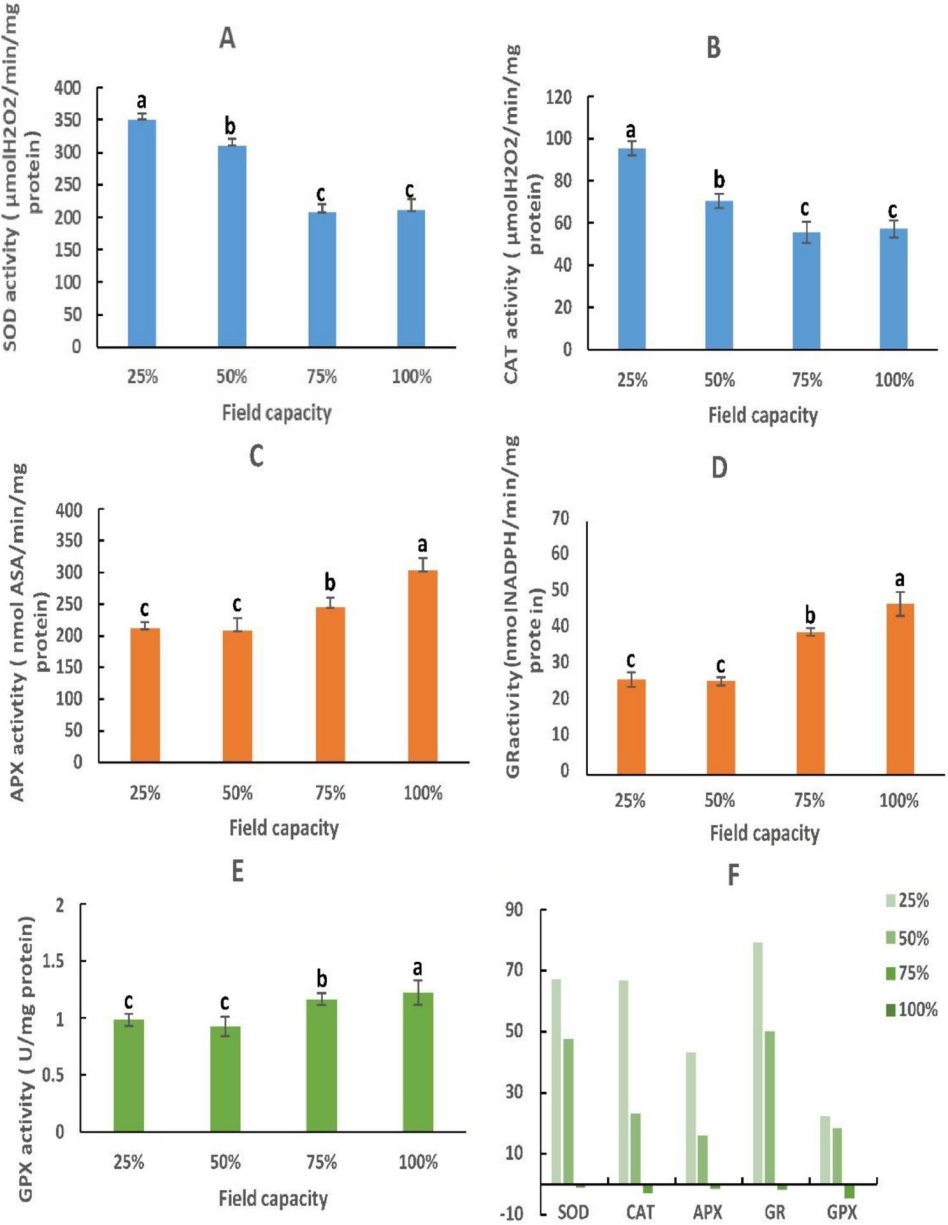
Relative (**A**, **B**, **C**, **D**, **E**) and percentage (**F**) changes of antioxidant enzyme activity: superoxide dismutase (SOD), catalase (CAT), ascorbate peroxidase (APX), glutathione reductase (GR), and glutathione peroxidase (GPX) activities in *M. oleifera* leaves submitted to different field capacities (25, 50 and 75%) compared to control condition (100%). Data represented as mean with error bars having different significant letters according to Tukey’s HSD test

Accordingly, our results (Fig. 3A-E) demonstrated that superoxide dismutase (SOD), catalase (CAT), ascorbate peroxidase (APX), glutathione reductase (GR), and glutathione peroxidase (GPX) activities were significantly stimulated. These increases were 76.15%, 66.74%, 43.26%, 79.30 and 22.2%, respectively in Moringa leaves stressed with 25% FC (Fig. 3F). Similar activations by stresses factors such as drought and salinity were demonstrated in other several studies [15, 51]. However, the astounding equilibrium between ROS production and antioxidative enzyme system activation reduces the detrimental effects of oxidative stress and increases the powerful ROS detoxifying capacity of the Moringa tree. This biochemical approach is an important tool for resisting, defending, and surviving in hard climate circumstances, such as abiotic stress.

### Inter-correlation among all data of M. oleifera trees under different field capacity regimes: multivariate analysis

Multivariate analysis approaches can be utilized to investigate correlations, categorization, and parameter prediction within complicated data sets since the results are more realistic, meaningful, and accurate [54].

#### Hierarchical co-clustering with heatmap

**Fig. 4:**
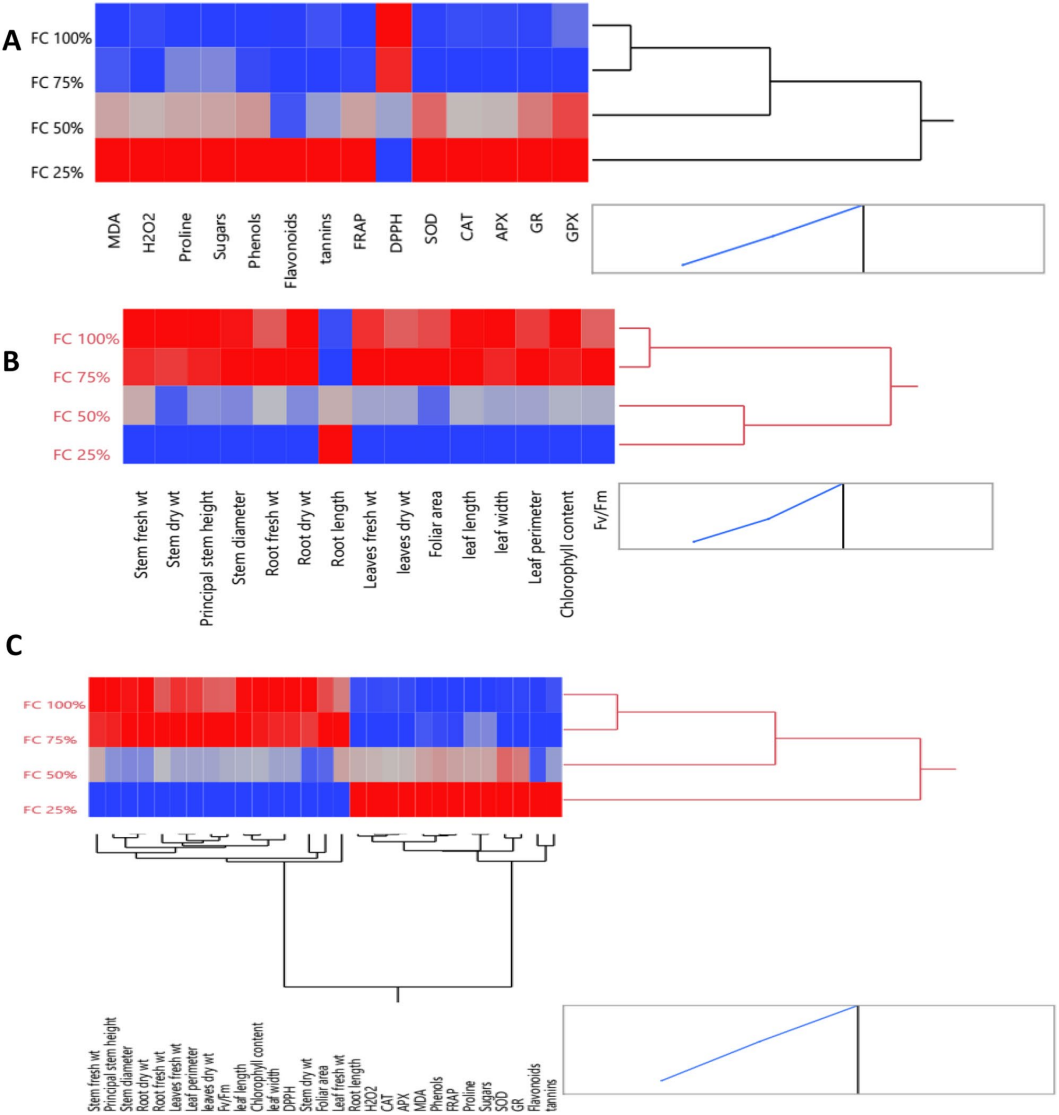
Multivariate analysis based on hierarchical dendrogram and heatmap correlation between (**A**) morpho-physiological parameters, (**B**) biochemical parameters and enzymatic and non-enzymatic antioxidants, (**C**) Two-way clustering using hierarchical co-clustering dendrogram and heatmap; row clusters were obtained at field capacity level, whereas the column cluster were recorded at trait or marker of all combined data of morpho-physiological, biochemical parameters and enzymatic and non-enzymatic antioxidants) of *M. oleifera* trees submitted to different field capacities (25, 50, 75 and 100%). Correlation levels are colored by red for positive correlation and blue for negative one

Multivariate analysis based on hierarchical co-cluster analysis using Ward’s method was performed to identify the distribution of the *M. oleifera* trees due to differences in morpho-physiological, biochemical and all combined data under different field capacities (25, 50, 75, and 100%) into two distinct clusters as represented in Fig. 4. Correlograms were based on the correlation coefficients of morphological and/or metabolic measurements. The red color indicated the positive correlation between measurements, whereas the blue color assumed the negative one.

In the presence of outlying observations in the dataset, the resilient hierarchical co-cluster (RHCOC) strategy generates a significantly lower clustering error rate than standard hierarchical clustering algorithms [55].

As shown in Fig. 4A, the hierarchical co-cluster dendrogram based on morpho-physiological parameters differences is divided into two clusters. The first cluster had *M. oleifera* trees under 25 and 50% FC and the second one is included *M. oleifera* trees under 75 and 100% FC. Under 75 and 100% FC, red positive correlation was found between all parameters except root length parameter (remarked as blue). Low water availability was favourable for the growth of Moringa plants. The plants displayed reduced height when they were fully capable of retaining water. In fact, reduced osmotic pressure during stressful situations, which causes root proliferation in quest of water and nutrients, may be the cause of the increase in root length at 70 and 40% water holding capacity [14].

At the same trend, the hierarchical co-cluster dendrogram based on biochemical and metabolic parameters differences is divided into two clusters. The first cluster had *M. oleifera* trees under 25% FC and the second one is divided into two sub-clusters. The sub-cluster I included *M. oleifera* trees under 75 and 100% FC and the other one contained 50% FC. Under 75 and 100% FC, DPPH is negatively correlated (remarked blue) with all other parameters (remarked as red) as illustrated in Fig. 4).

A two-way co-cluster matrix using hierarchical co-clustering dendrogram and heatmap; row clusters were obtained at field capacity level, whereas the column cluster were recorded at trait or marker of all combined data of *M. oleifera* trees submitted to different field capacities (Fig. 4C). The co- cluster was consisted of 4 rows (treatments) and 29 column clusters (traits). At the row clustering (FC level), it is divided to two clusters. The first cluster had *M. oleifera* trees under 25% FC and the second one is classified into subclusters I and II. The subcluster I had *M. oleifera* trees under 50% FC and the other subcluster II included *M. oleifera* trees under 75 and 100% FC. At the column clustering (parameters), it is divided into two major clusters. The first cluster had the following parameters: root length, H_2_O_2_, CAT, APX, MDA, phenols, FRAP, proline, sugars, SOD, GR, flavonoids, and tannins. On the other hand, the second cluster had stem, root, leaf fresh and dry weight, stem diameter and height, leaf length, width, foliar area, chlorophyll content, Fv/Fm and DPPH. Sun et al. [56] reported progressive drought stress generally caused an elevated in activity of SOD, POD, CAT, and APX enzymes that coordinate ROS concentration.

#### Scatter plot matrix with correlations circles

**Fig. 5:**
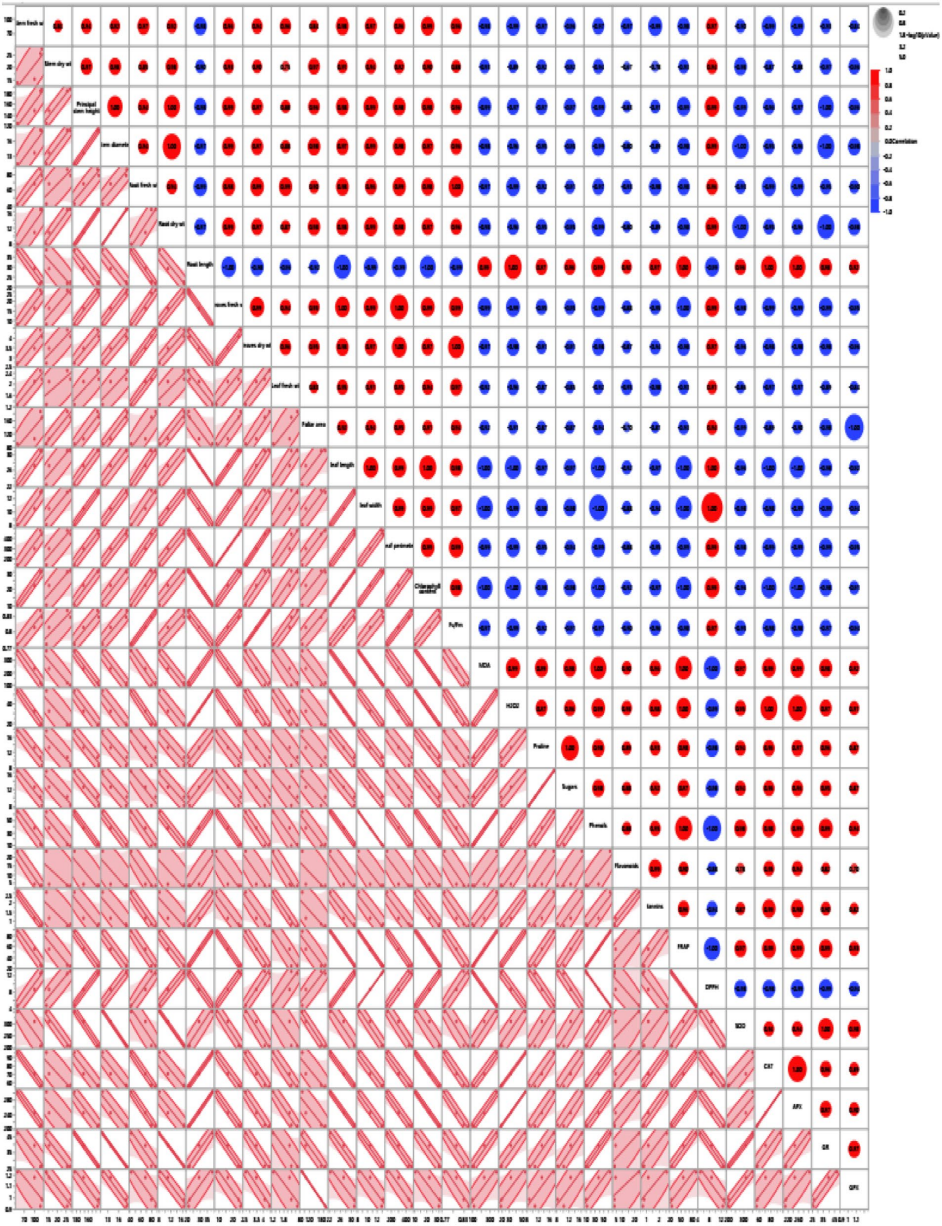
Scatter plot matrix with heatmap correlation circles of all combined data (morphological, physiological, and metabolic measurements) of *M. oleifera* trees submitted to different field capacities (25, 50, 75 and 100%). Red color indicates positive correlation, blue colour indicates the negative correlation, while the size of circles indicates the significance (see scale at the above right corner)

Scatter plot matrix with heatmap correlation circles of 29 quantitative traits (morphological, physiological, and metabolic measurements) of *M. oleifera* trees submitted to different field capacities (25, 50, 75, and 100%). The scatter plot matrix showed the density ellipses in each individual scatter plot and the red circles contain about 95% of the data as represented in (Fig. 5). Red color assumed the positive correlation, blue colour assumed the negative correlation, while the size of circles indicated the significance. In this investigation, root length was negatively correlated with all morpho-physiological parameters, but it was positively correlated to all biochemical and metabolic parameters except DPPH. Highly positive correlation was detected among root length, MDA, H_2_O_2_, phenols and FRAP.

On contrary, DPPH was positively correlated to all morpho-physiological parameters except root length and was negatively correlated with all biochemical to FRAP, MDA and phenols. In the same trend, MDA was strongly positive correlated with H_2_O_2_, proline, phenols and FRAP. Also, Fv/Fm showed the maximum positive correlation to chlorophyll content, and it also correlated positively with all leaf parameters. The adaptive response of Moringa to drought stress caused by strong accumulation by endogenous proline and an increase in the total polyphenol and radical scavenging activity. A strong correlation of these compounds’ accumulation and higher radical scavenging activity against FRAP and DPPH radical were detected confirming their role in antioxidant defense potential of Moringa [15, 51].

Concerning the enzymatic antioxidants, all enzymes were positively correlated with each other. CAT was highly positive correlate to APX and H_2_O_2_ in the present study. Plants that are tolerant to drought may display modifications in their cellular metabolism as a defense against oxidative damage. The principal enzymes that eliminate H_2_O_2_ from leaves are SOD, CAT and APX. While both CAT and APX enzymes work together, APX is more probable to bind to H_2_O_2_ than CAT does. The moderate stressed plants displayed elevated enzymes activities (SOD, CAT, and APX) at the first maximum stress; only the SOD activity persisted as elevated at the second maximum stress. Peroxisomes and chloroplasts, the primary sites of action of APX, may have provided enhanced protection because of this treatment [53].

#### Principal component analysis (PCA) biplot

Principal component analysis (PCA) is used to analyze and deconstruct large, complex datasets. The only PCAs deemed significant were those with eigenvalues larger than one. The item diversity was analyzed in terms of quality features using the PCA technique, and the objects were grouped based on the similarity hierarchy [57]. PCA-biplot is one of the most efficient multivariate techniques for evaluating trait interaction and genotypic performance, and it is widely used to investigate trait association in various agricultural plants [58].

**Fig. 6:**
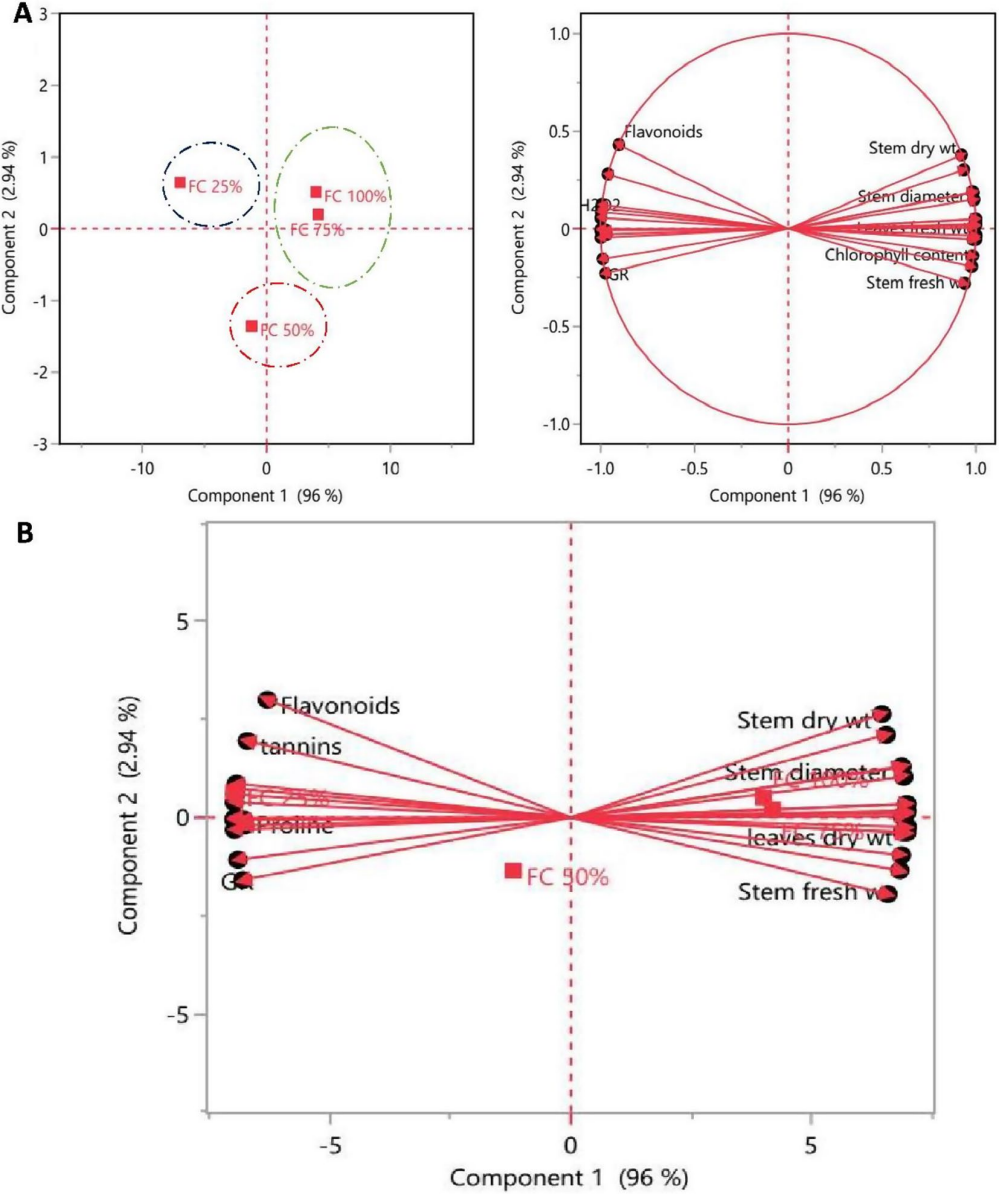
Principal component analysis (PCA), (**A**) the PCA scatter plot, and (**B**) biplot illustrating the distribution of different field capacity regimes on *M. oleifera* trees, on PC1 and PC2 components based on the analysis of all combined data (morphological, physiological, and metabolic measurements). Blue, red and green dashed rings showed three different clusters. The dots were *M. oleifera* trees under different regimes of FC, and the vectors (red arrows) were parameters. The abbreviations were previously mentioned in the earlier figures

In a PCA scatter plot, *M. oleifera* trees submitted to different field capacities (25, 50, 75, and 100%) described by the first 2 PCs per cluster. The first component PC1 was recorded as 96% and the second component PC2 as 2.94% of the total variation. The studied treated *M. oleifera* trees were also grouped into three clusters: cluster I (green group) had treated *M. oleifera* trees subjected to 75 and 100% FC, cluster II (blue group) had *M. oleifera* trees subjected to 25% FC and cluster III (red group) had *M. oleifera* trees subjected to 50% FC which were distinct and well supported as illustrated in Fig. 6A.

In the system of the two first components, length of vector and cosine of angle were used for discrimination of treated *M. oleifera* trees. The small angle between these vectors: stem dry weight, foliar area, root dry weight, leaves fresh weight, stem fresh weight, Fv/Fm proves a significant strong positive correlation between these traits and were found as the most effective parameters in differentiation between *M. oleifera* trees under 75 and 100% FC. On the other hand, flavonoids, tannins, sugars, CAT, SOD, GR were found as the most effective parameters in differentiation between *M. oleifera* trees under 25% FC. Flavonoids and tannins had an acute angle which indicated highly positive correlation to each other. In the same manner, GR with SOD and GPX and proline with phenolic compounds Fig. 6 B. The multivariate analysis confirmed the correlation data reported before.

Previous studies have successfully evaluated drought tolerance indicators for wheat [59] and cotton [60] by combining principal component analysis and/or regression analysis. Also, Wang et al. [61] examined 12 physiological indicators related to wheat drought tolerance, including photosynthetic characteristics (Pn, Gs, Fv/Fm, qP, NPQ, and qN), SOD activity, and MDA content, as well as osmoregulatory substances (Pro, SS, HXKs, and GLC), were selected to further screen physiological key indicators to evaluate drought tolerance in wheat. In maize, Balbaa et al. [62] used discriminative analyses such as PCA to support their methodology as an unambiguous differential approach, indicating that chlorophyll content and transpiration rate traits were significant on maize inbred line performance under stress conditions, while other remained traits were most discriminatory under normal conditions.

## Conclusion

Water stress is on the rise these days due to changes in the global climate, which is a major issue that is detrimental to plant cover. *Moringa oleifera* trees demonstrated the ability to tolerate this type of abiotic stress, responding in a number of ways that were responsive to the water-deficit state. To withstand the conditions of drought stress, root morphological modifications compensated the limitation of tree shoot development. First and foremost, it is important to emphasise that *M. oleifera’s* ability to adjust to the water unavailability conditions (25, 50, 75 and 100% field capacities, it was subjected to was evidenced by its regression of growth, the dimensions of its aerial organs and the elongation of its roots. Furthermore, we have found important connections between the activation of the defence antioxidant system (superoxide dismutase (SOD), catalase (CAT), glutathione reductase (GR), ascorbate peroxidase (APX) and glutathione peroxidase (GPX) enzymes), hyper-accumulation of secondary metabolites (phenolic compounds, total flavonoids and condensed tannins) and osmoregulators (proline and soluble sugars), and osmotic stress brought on by a lack of water. A powerful antioxidant radical scavenger (FRAP and DPPH) that was enhanced by shortage water was another important component of stress adversity adoption. Furthermore, *M. oleifera* shows potential as an ecological solution for agroforestry systems, particularly in dry locations where water scarcity is an issue. Further research can explore the ability of different Moringa parts to stimulate plant growth under varied water stress scenarios.

### Electronic supplementary material

Below is the link to the electronic supplementary material.

Supplementary Material 1

## Data Availability

Data is provided within the manuscript or supplemetary information files.
