# Supplementary material for: Multivariate investigation of Moringa oleifera morpho-physiological and biochemical traits under various water regimes
Source: BMC Plant Biol. 2024 Jun 6;24:505. doi: 10.1186/s12870-024-05040-5 (PMC11155125; doi:10.1186/s12870-024-05040-5)
Supplement: Supplementary file 1 — Supplementary Material 1 [file 12870_2024_5040_MOESM1_ESM.docx]

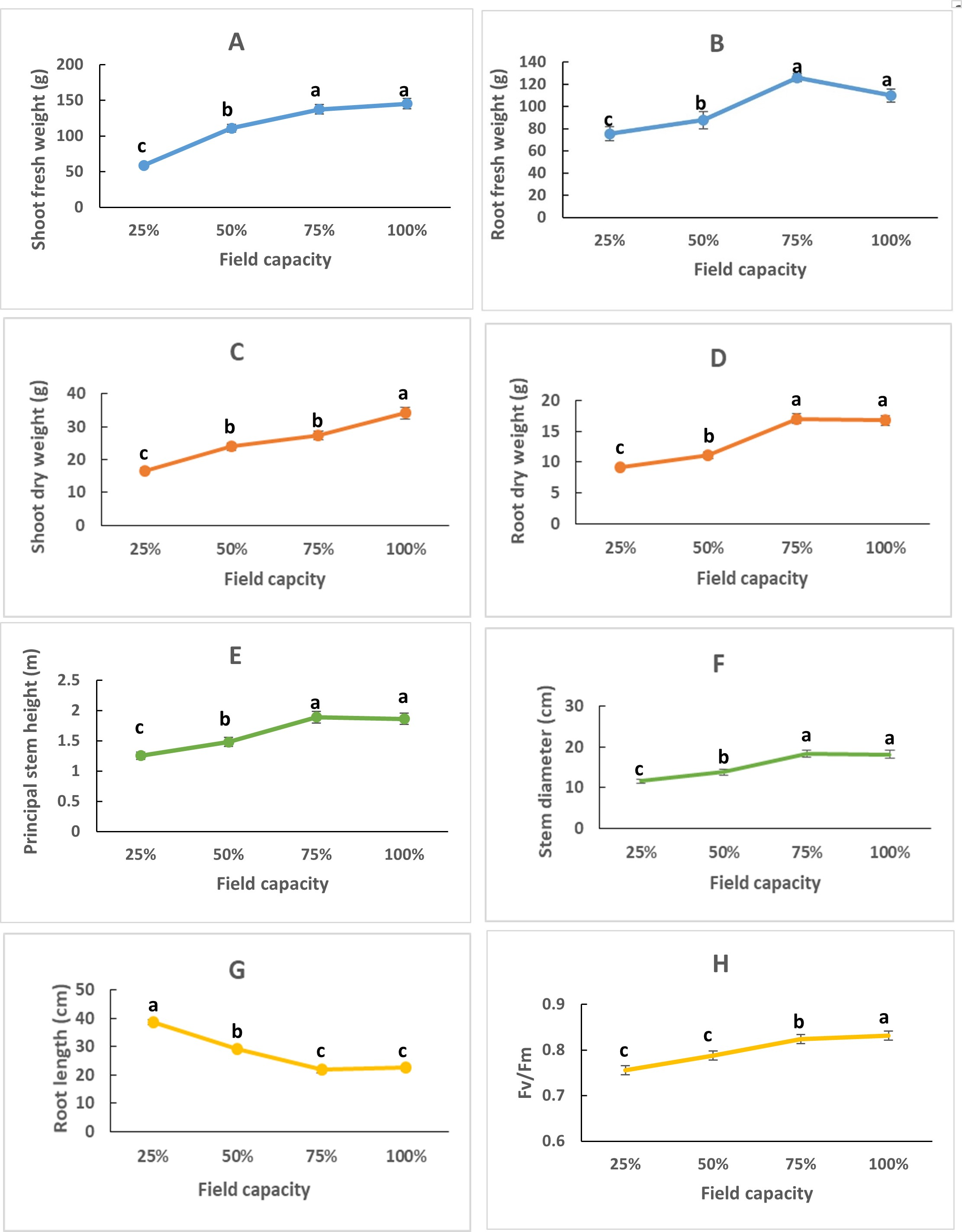


**Supplementary Figure 1.** Changes of shoots and roots fresh and roots weight, stem height and diameter, root length and PSII efficiency (Fv/Fm) of *M. oleifera* trees submitted to different field capacities (20, 50, 75 and 100%). One way ANOVA represented as error bars with the superscript letters showing statistically significant differences (according to Tukey’s HSD test) at *P* ≤ 0.05.


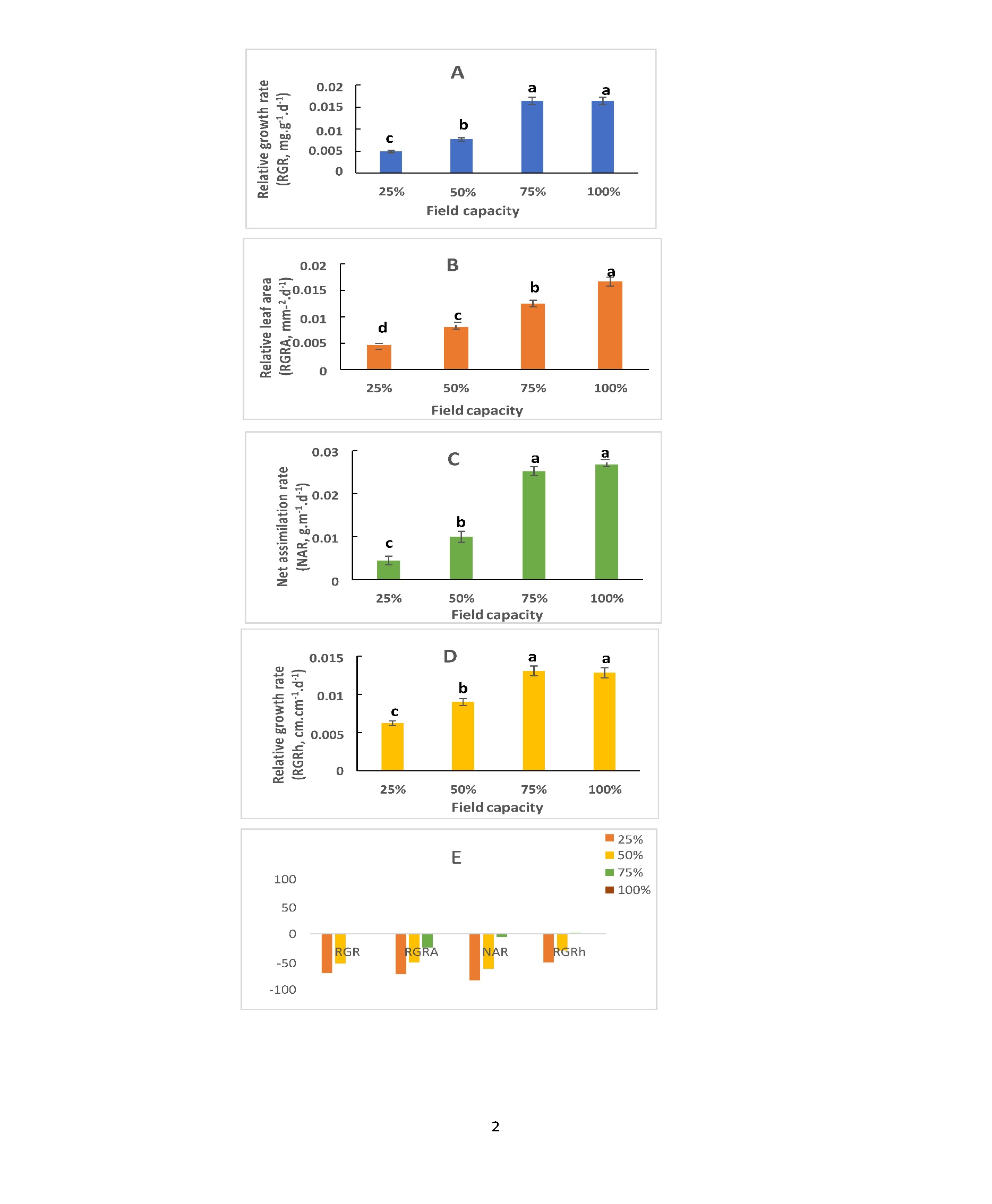


**Supplementary Figure 2**. Relative (A, B, C, D) and percentage change (E) of growth rates of *M. oleifera* trees submitted to different field capacities (25, 50, 75 and 100%). One way ANOVA represented as error bars with the superscript letters showing statistically significant differences (according to Tukey’s HSD test) at *P* ≤ 0.05.
